# Boosted visual performance after eye blinks

**DOI:** 10.1167/jov.20.10.2

**Published:** 2020-10-01

**Authors:** Jit Wei A. Ang, Gerrit W. Maus

**Affiliations:** Psychology Programme, School of Social Sciences, Nanyang Technological University, Singapore; Psychology Programme, School of Social Sciences, Nanyang Technological University, Singapore

**Keywords:** eye blinks, attention, default mode network, attention network

## Abstract

We blink more often than required for maintaining the corneal tear film. Whether there are perceptual or cognitive consequences of blinks that may justify their high frequency is unclear. Previous findings showed that blinks may indicate switches between large-scale cortical networks, such as dorsal attention and default-mode networks. Thus, blinks may trigger a refresh of visual attention. Yet, this has so far not been confirmed behaviorally. Here, we tested the effect of blinks on visual performance in a series of rapid serial visual presentation tasks. In Experiment 1, participants had to identify a target digit embedded in a random stream of letter distractors, presented foveally for 60 ms each. Participants blinked once during the presentation stream. In a separate condition, blinks were simulated by shutter glasses. Detection performance was enhanced (up to 13% point increase in accuracy) for targets appearing up to 300 ms after eye blinks. Performance boosts were stronger for voluntary blinks than artificial blinks. This performance boost was also replicated with more naturalistic stimuli (Experiment 2). We conclude that eye blinks lead to attentional benefits for object recognition in the period after reopening of the eyelids and may be used strategically for temporarily boosting visual performance.

## Introduction

We blink more often than required for maintaining the corneal tear film. Blink frequencies fluctuate greatly depending on task. For instance, people tend to blink more frequently when they are engaged in a conversation and less during reading (Bentivoglio et al., 1997). These fluctuations of blink frequencies are unlikely due to different requirements for cornea lubrication during different cognitive states. The precorneal tear film starts drying up on average 25 s after a blink (Norn, 1969). This would suggest that two to three blinks per minute would be enough to maintain the tear film.

More plausibly, different blink rates reflect different mental processing states. For example, reduced blink frequencies during reading (Bentivoglio et al., 1997) might be attributed to the increased visual bandwidth required for efficient absorption of information. However, the benefits of increased blink frequencies during conversations are less apparent. What could be the benefit of increased blink rates in certain cognitive or perceptual scenarios? Do blinks trigger other physiological responses with consequences for perception or cognition?

Blink frequencies could reflect neural activity in major cognitive neural networks. For example, the dorsal attentional network (DAN) and the default mode network (DMN)—respectively associated with externally motivated attention-demanding tasks (Vanhaudenhuyse et al., 2011) and internal mental processes such as mind wandering (Mason et al., 2007)—are anticorrelated in their activities (Fox et al., 2005). A recent study found eye blinks to be associated with activation of the DMN and simultaneous deactivation of the DAN when blinks were measured while participants viewed a movie clip (Nakano, Kato, Morito, Itoi, & Kitazawa, 2013). Interestingly, blinks tended to occur at specific and consistent time points during the movie across participants (Nakano, Yamamoto, Kitajo, Takahashi, & Kitazawa, 2009). Blinks may reflect transitions in cognition, during which previously attended visual information is consolidated and processed by the internal cognition-oriented DMN. Each blink might thus indicate the onset of a new episode of “refreshed” attention.

Blinks are costly to perception. The eyelids cover the pupil for a period of up to 300 ms (Stern, Walrath, & Goldstein, 1984), impairing spatial information at the retina and introducing strong global transient signals. At typical blink rates, about 10% of overall time is spent with the eyes closed (Lawson, 1948). Yet, the disruptions caused by spontaneous eye blinks are typically not perceived. Behavioral experiments suggest that the transients induced by eye blinks are suppressed. Light flashes presented through the roof of the mouth to bypass the pupil and eyelids are less detectable when they coincide with eye blinks (Manning, Riggs, & Komenda, 1983; Riggs, Volkmann, & Moore, 1981; Volkmann, Riggs, & Moore, 1980).

Blinks trigger neural activity in the posterior parietal cortex (Hari, Salmellin, Tissari, Kajola, & Virsu, 1994). This activity did not occur, however, for blinks made in complete darkness and was therefore hypothesized to be related to maintaining perceptual continuity throughout the blink. Conversely, a functional magnetic resonance imaging (fMRI) study found suppression of activity in visual areas: Stimulation of the retina with a flashing light through the roof of the mouth exhibited reduced activity in parietal as well as frontal regions when participants blinked as compared to no blinking (Bristow, Haynes, Sylvester, Frith, & Rees, 2005). Electrocorticography measures during visual stimulus presentations showed a drop of neural activity in higher ventral visual regions when participants observed brief screen blanking, followed by an immediate rebound activity that overshot baseline activity (Golan et al., 2016). This response drop was attenuated for blinks. These studies show that extraretinal signals (e.g., motor signals signifying the execution of a blink) seem to reduce transients resulting from the blink, which may result in the lack of awareness of the abrupt visual disruption and thus allow for perceptual continuity across a blink.

To look for potential cognitive benefits of eye blinks, we investigated the effect of eye blinks on perception and attention. We used a modified rapid serial visual presentation (RSVP) task. Participants were asked to identify a single target that was embedded at a random time point in a rapid stream of successive distractor stimuli. In three conditions, viewing was interrupted by a voluntary eye blink, interrupted by an artificial blink caused by shutter glasses, or remained continuous throughout the presentation. In three experiments with different stimuli, we evaluated target identification performance as a function of presentation time after voluntary and artificial blinks and compared this to performance in control conditions without blinks. Participants were asked to identify a target digit embedded in letter distractors (Experiment 1) or identify an animal target embedded in scene distractors (Experiments 2A, 2B).

Blink-induced suppression (Bristow et al., 2005; Golan et al., 2016; Volkmann et al., 1980) may predict that visual performance is affected right after a blink. In contrast, a “refresh” of attention induced by eye blinks (Nakano et al., 2013) may predict better performance just after a blink.

## General methods

### Participants

Seventy-eight participants (30 men, age range: 18–33 years, mean: 23.8 years, *SD*: 3.9 years) were recruited from Nanyang Technological University and received course credits or S$10 per hour for their participation. Experiments 1 and 2A had 32 participants. Experiment 2B had 14 participants. All participants had normal or corrected-to-normal visual acuity (only contact lenses were used for correction to allow for use of the occlusion goggles). Informed consent was obtained from all participants. The study was approved by the Institutional Review Board of Nanyang Technological University.

### Apparatus

Stimuli were presented on a 21-in. CRT monitor (FD Premium; SUN Microsystems, Santa Clara, California, United States) with a screen resolution of 1,152 × 864 at a refresh rate of 100 Hz. Participants sat 58 cm from the monitor with their heads rested on a chinrest. Stimuli were generated using the PsychoPy software package (Peirce, 2007). Eye blinks were detected in real time using an Eyelink 1000+ (SR Research, Ottawa, Canada). A 5-point monocular calibration procedure was used to calibrate the eye tracker to each participant's right eye. To simulate eye blinks artificially, participants wore Plato Occlusion Goggles (Translucent Technologies, Toronto, Canada), controlled by the stimulation software via an Arduino (Strambino City, Turin, Italy) microcontroller board.

### Procedure

**Figure 1.**
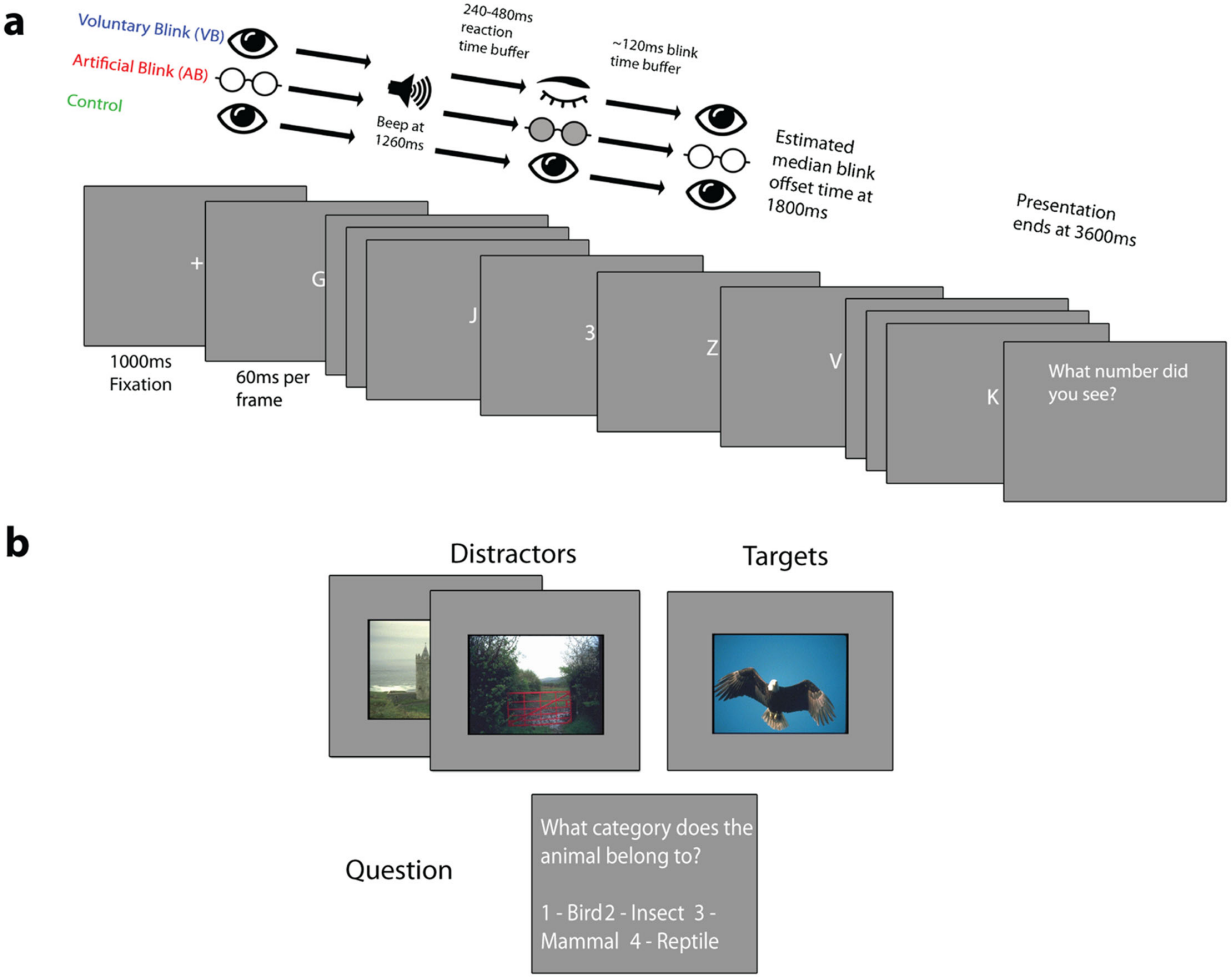
Experimental paradigm. Stimuli and procedure of the RSVP task. (A) In Experiment 1, a single target digit was embedded in a rapid stream of distractor letters, each frame presented for 60 ms. In the VB condition, the target could be presented at a random time before or after the end of a voluntary blink. In AB, occlusion goggles simulated a blink, and the target was shown before or afterward, accordingly. In the control condition, the target appeared randomly at any time during the trial (except the first and last five frames). (B) In Experiments 2A and 2B, the trial sequence was the same as in Experiment 1, but animal targets and scene distractors were used instead.

We presented participants with a single target stimulus embedded in a stream of distractor stimuli. The precise nature of the stimuli was different in each experiment and is described in Figure 1. Each trial started with 1,000 ms of fixation before the RSVP stream commenced. Each frame in the stream was presented for 60 ms (six refresh frames of the monitor).

In the control condition, the target appeared at a random frame of the trial, excluding the first and last five frames. At the end of each trial, participants were asked to indicate their response via a key press.

In the voluntary blink condition (VB), target appearances were random across trials (excluding only the first and last five frames of the sequence) independent of the timing of the blink. Participants were asked to blink their eyes when they heard an auditory 50-ms beep (432-Hz pure tone) that was played through stereo speakers after 1,260 ms from trial onset. This would on average lead participants to blink somewhere mid-trial (total trial duration 3,600 ms). Experiment 2B allowed participants to initiate the artificial blink by pressing the space bar. Table 1 summarizes the differences between all experiments.

**Table 1.** Experimental variations. *Notes:* Summary of the conditions in all experiments. The table lists what targets and distractors consisted of in each experiment, as well as how voluntary or artificial blinks were triggered.

Experiments	Target/distractor	Voluntary blink	Artificial blink	Control
1: Digit identification	Digit/letter	Cued by tone	Automatic after tone	Eyes open
2A: Animal identification	Animal/scene	Cued by tone	Automatic after tone	Eyes open
2B: Animal identification	Animal/scene	Cued by tone	Self-initiated after tone	Eyes open

Trials in which blinks occurred after the target were also classified as control. Trials with more than one blink before target appearance or with excessive noise in the tracking data were discarded.

In the artificial blink condition (AB), the sequence followed VB, except that blinks were simulated by closing the occlusion goggles. For Experiments 1 and 2A, the occlusion was triggered to occur at a random time between 1,500 and 1,740 ms into the trial (after the beep cue at 1,260 ms). The occlusion for Experiment 2B was manually triggered by the participants after the tone. The occluding duration was set to each participant's mean individual blink duration (± 1 *SD*) measured from a resting baseline blink rate (see below).

### Baseline resting blink duration

Before the main experiment, we measured baseline blink durations for each participant to adjust the duration of artificial blinks for each participant. Participants were asked to relax, do nothing, and fixate a white cross on a black background on the monitor screen for 3 min, while the eye tracker was used to record blinks. Each individual participant's blink duration (± 1 *SD*) was used to determine their range of artificial blink durations in the subsequent experiments.

The blink durations measured with our eye tracker (*M* = 147.8 ms, *SE* = 0.55 ms) are comparable to published values (Choi, Park, Sung, & Kim, 2003; Kwon et al., 2013; Sun et al., 1997).

### Analysis

We plotted average target identification performance (percent correct) as a function of target presentation time after the end of a blink (for VB and AB). Data were plotted in bins of 60 ms, with each bin smoothed by a rolling-average window of 120 ms (± 60 ms). As targets were scattered over many times bins with limited trials, data from all participants were collapsed together to reach a sufficient number of trials for all time bins. The average performance in no-blink control conditions is plotted for comparison. Similar results were found when we repeated the analysis with different bin spacings or averaging window sizes (see Supplementary Figure S1).

As there are many time bins (total trial duration divided by 60 ms) to compare across conditions, we opted to use a bootstrap method to make inferences about the performance of the population from the sample data. Here, each bin's performance was compared to the no-blink control condition (control data collapsed across two sessions) and the other conditions using bootstrap tests. We first resampled data from the collapsed data set with replacement over 10,000 iterations. For each iteration, performance was then binned (and smoothed) in 60-ms bins. Bins with significant differences between conditions were detected if bootstrapped replacement samples led to better performance in one condition over another condition on more than 97.5% or less than 2.5% of the 10,000 iterations (two-tailed *p* ≤ 0.05). We also calculated the bootstrap standard error (standard deviation of the bootstrap distribution).

The *p*-values were corrected for multiple comparisons using the false discovery rate (FDR) Benjamini-Hochberg (BH) procedure, with the threshold value set at 0.05. First, the *p*-values of all comparisons were consolidated into a list, ranked in ascending order. This was done individually for all comparisons (VB vs. control, AB vs. control, and VB vs. AB). Next, the BH critical value was generated for each of the bootstrap *p*-values using the following formula:

$$\mathrm{BH}\;\mathrm{critical}\;\mathrm{value}=\left(i/m\right)Q,$$

where *i* is individual *p*-value rank within each list, *m* is the total number of comparisons, and *Q* is the FDR of 0.05. Bootstrap *p*-values that fall below the corresponding BH critical value were deemed to be statistically significant.

Performance data before the blink were not evaluated as they were not the primary focus of the current study.

## Experiment 1: Number identification task

### Methods

We tested the effect of voluntary and artificial blinks on target identification performance in an RSVP task with a number digit target in a sequence of letter distractors (Figure 1A). Distractor stimuli consisted of single capital letters from the Roman alphabet (Arial font) presented in the center of the screen, subtending 0.69 degrees of visual angle (vertical extent). Targets consisted of single Arabic numeral digits (Arial font), omitting digits 0, 1, and 2 due to their similarity to O, I, and Z, respectively, which were also omitted from the distractor list. All stimuli were white (132 cd/m^2^) presented on a gray (66.4 cd/m^2^) background. Each stimulus frame in the sequence was shown for 60 ms.

The timing of the target was fully randomized to occur at any point in the trial. Blinks were cued by a beep. Trials lasted for 60 frames (3,600 ms). The beep occurred at 1,260 ms, so that the end of the subsequent blink would be approximately halfway through the trial. In the AB condition, the glasses shut at a random delay 240 to 480 ms after the beep. Targets could occur at any frame during the trial apart from the first and last five frames (300 ms). The experiment consisted of 6 blocks of 50 trials for VB and AB with one additional block of control condition in each session, for a total of 14 blocks per participant over two sessions.

### Results

**Figure 2.**
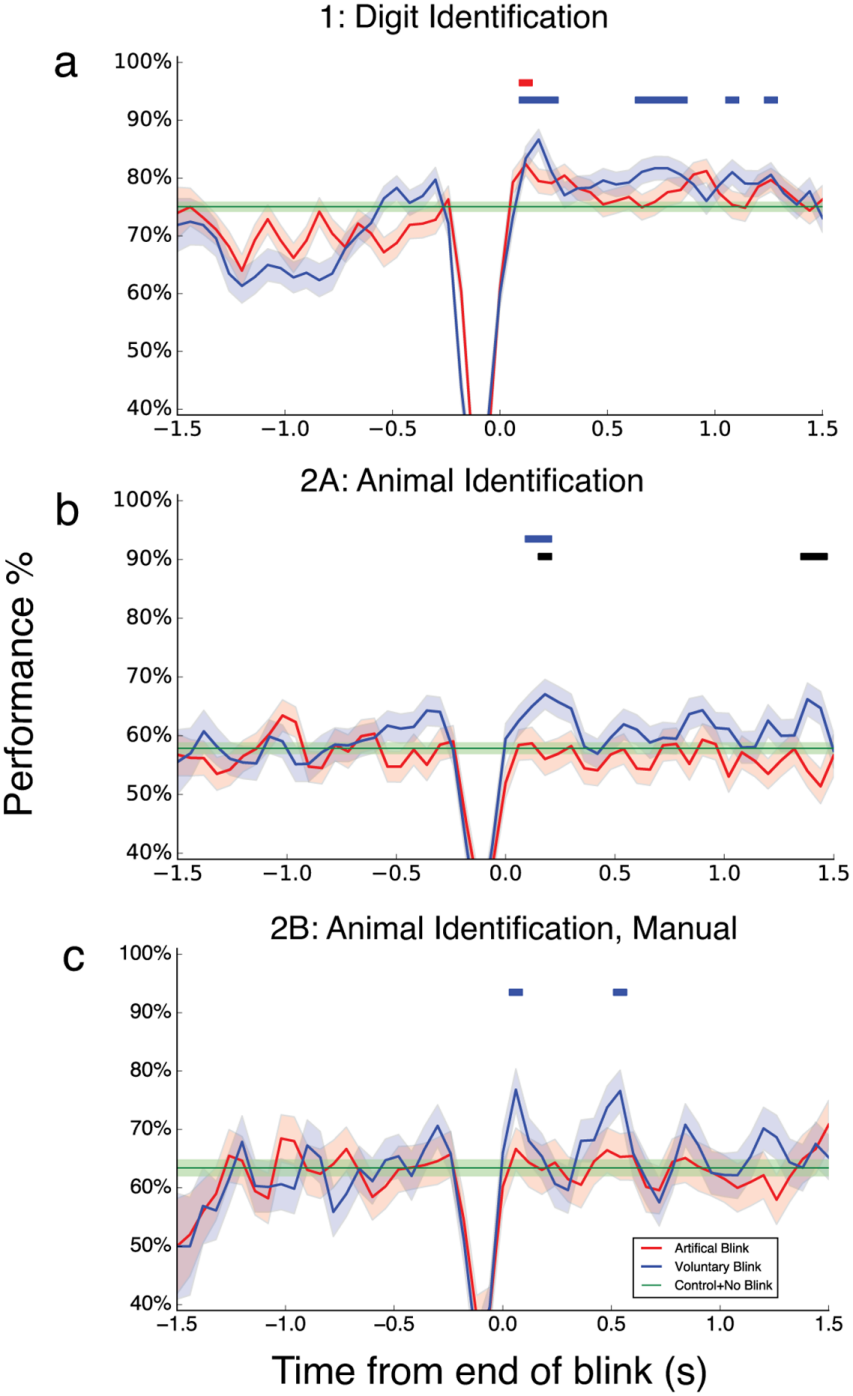
Results. Task performance as a function of target presentation time after voluntary and artificial blinks for all experiments. Responses were binned in 120-ms bins, plotted every 60 ms, time-locked to the end of the blink. Trials with targets appearing up to 1.5 s before and after the blink are shown. The grand average performance in the no-blink control condition is plotted as a horizontal line (green) for easy comparisons. Shaded areas are bootstrap standard errors. Horizontal lines at the top show periods after the blink with significant differences (*p* < 0.05 after correcting for multiple comparisons) from the respective control condition (blue and red) or between VB and AB (black). (A) Experiment 1 (*N* = 32), (B) Experiment 2B (*N* = 32), and (C) Experiment 2C (*N* = 14).

Performance for the no-blink control condition was on average 75.0% (*SE* = 0.8%) higher than the chance rate of 14.3%. Note that all standard errors reported in this article are bootstrap standard errors (see General Methods). Figure 2A shows performance in the control condition (horizontal green lines) and for the VB and AB conditions as a function of target time before and after a blink. We found a significant boost in the VB condition after the blink up to ∼300 ms (Figure 2A), peaking at ∼180 ms (86.7%, *SE* = 1.8%). AB peaked early at ∼120 ms (82.4%, *SE* = 1.9%), but this boost was less pronounced and sustained as compared to VB. Later significant boosts for VB were also observed at ∼600 to 900 ms (peak: 83.2%, *SE* = 3.2%), ∼1,080 ms, and ∼1,260 ms.

The performance boost is unlikely due to the beep cue: When performance is plotted as a function of time, locked to the beep onset, performance did not consistently spike after the beep (Supplementary Figure S2).

While we did not analyze performance before the blink systematically, it is interesting to note the apparent decrease in performance before voluntary blinks in Experiment 1. This may be due to the implicit attentional demands toward the upcoming blink audio cue, resulting in decreased attention to the visual stream while awaiting the cue to blink. Alternatively, the decrease in performance may reflect motor planning processes before the execution of a blink. However, Experiments 2A and 2B did not result in a similarly apparent performance decrease before blinks, precluding any definite conclusions.

In summary, Experiment 1 found a boost in target recognition performance immediately after eye blinks. Artificial blinks with shutter glasses also led to better performance right after the opening of the glasses. However, this boost is mild, suggesting that voluntary eye blinks elicited a stronger boost in attention performance.

Some of the boost may, in part, be an effect of the visual transient of reopening of the eyelids (or shutter glasses). But voluntary blinks (also in subsequent experiments) produced an even stronger effect compared to artificial blinks. At least, it should be noted that eye blinks could be used strategically to generate such a boost at time points, when temporal attention is required (Hoppe, Helfmann, & Rothkopf, 2018). The performance boost might also be a consequence of activity switches between large-scale neural networks (Nakano et al., 2013).

## Experiments 2A, 2B: Naturalistic stimuli, animal identification task

To assess whether the results from Experiment 1 are robust to the kind of stimulus and task used, we employed a naturalistic stimulus set in Experiment 2. Stimuli consisted of photographs of natural scenes, with target photos containing an animal. The task was to detect whether an animal was present and to identify the category that the animal belongs to (Figure 1B).

### Methods

Stimuli were taken from a published image data set (Li, VanRullen, Koch, & Perona, 2002), including a collection of 1,088 natural scenes as distractors and 24 target images, consisting of six animals from each of four categories (birds, insects, mammals, and reptiles). Random distractor images were presented consecutively for 60 ms each at a size of 600 × 400 pixels (19.76° × 13.47°) centered on the screen on a gray background.

As with Experiment 1, timing of target appearance was completely randomized across trials apart from appearing in the first or last five frames. Experiment 2 aimed to independently replicate the finding on Experiment 1 on a different set of stimuli.

Experiment 2B is mostly identical to Experiment 2A. The only difference is that the shutter glasses were manually triggered by the participants. Participants were told to press the space bar immediately after hearing the beep tone, which, in turn, initiated the artificial blink with no delay. This was to rule out the possibility that a lack of voluntary initiation of blinks in the artificial blink conditions confounded the result.

### Results

For Experiment 2A, performance on the categorization task in the control condition was above chance level (25%), mean = 57.9%, *SE* = 0.9% (Figure 2B). VB led to increased performance from ∼60 to 240 ms after the blink, reaching peak performance of 67.1% (*SE* = 2.6%) correct at ∼180 ms, showing significant differences to both control and AB performance. VB performance also peaked again at ∼1,380 ms (mean = 64.3%, *SE* = 2.5%) (Figure 2B). AB performance did not differ from baseline performance at any time points (other than during the closure of the shutter glasses, when performance obviously dropped).

For Experiment 2B, control performance was above chance (63.4%, *SE* = 1.3%) (Figure 2C). VB performance of 2A was replicated in 2B, where there was a significant boost after the eye blink to ∼120 ms, peaking at ∼60 ms (mean = 76.8%, *SE* = 3.6%). There was also a second boost observed at ∼540 ms (mean = 76.6%, *SE* = 3.5%). No boost was observed in the AB condition.

In summary, the general pattern of results from Experiment 1 was replicated in Experiment 2 for a completely different set of stimuli. Like in Experiment 1, there was a significant performance boost immediately after the end of a blink. This boost from voluntary blinks was evident in both Experiments 2A and 2B, with no boost observed from artificial blinks, indicating that eye blinks have effects on visual performance over and above their optical effects. In Experiment 2B (and Experiment 1), we also found more sustained performance improvements in later periods after voluntary blinks. This boost effect is unlikely to be confounded by the lack of active initiation of the blank in artificial blink conditions as the effect persists in Experiment 2B, in which artificial blinks were triggered by a key press.

## Discussion

RSVP paradigms, as employed here, are oftentimes used in the study of the “attentional blink,” where the ability to detect a second target, which appears shortly after the first target, is very poor (Pashler, 1994; Raymond, Shapiro, & Arnell, 1992). Here, there was only one target, and we studied the effect of *real* eye blinks—rather than “blinks” of attention—on visual performance.

We hypothesized that changes in large-scale network activity after eye blinks (Nakano et al., 2013) might lead to an “attentional refresh” and thus improve perceptual performance immediately after blinks. However, suppression of blink-induced transients (Bristow et al., 2005; Golan et al., 2016; Volkmann et al., 1980) might also be expected to impair perceptual performance immediately after blinks. We found an early boost in performance immediately after eye blinks lasting for up to ∼300 ms in object recognition tasks with simple letter and number (Experiment 1) and more complex natural stimuli (Experiment 2). Two experiments (Experiments 1, 2B) also showed signs of a second and more sustained later boost after the end of a blink.

The early boost for object recognition may in part be stimulus driven, as artificial blinks produced by occlusion goggles led to a some early boost as well (Experiment 1), akin to attention-grabbing sudden stimulus onsets (Yantis & Jonides, 1984). This initially suggests that eye blinks can facilitate object recognition but in the form of a physical aid rather than a cognitive boost. By removing the visual input for a fraction of a second, the blink reduces forward-masking effects (Breitmeyer, Hoar, Randall, & Conte, 1984) in the RSVP stream. However, in Experiment 2, only voluntary blinks enjoyed significant performance boosts after eye blinks. Also, the performance boosts after voluntary blinks were stronger and lasted longer than after artificial blinks. This suggests that while abrupt appearance of stimuli is attention grabbing, voluntary blinks provided an additional attentional boost on top of the boost contributed by the sudden appearance of stimuli itself.

These performance boosts may be consistent with changes in large-scale cortical network activity triggered by eye blinks, as reported in an earlier fMRI study (Nakano et al., 2013). While the exact timing of these neural events is hard to determine with fMRI methods only, we propose that the behavioral boost found in the present study could be the consequence of newly refreshed attention following the blink-induced release of attention. Electrophysiological measures may be better suited to look for neural signatures of this effect in the future.

Alternatively, our results might be related to a phase reset of attentional rhythms. Previous studies using visual detection paradigms have identified attentional rhythms, oscillating at about 4 Hz (Landau & Fries, 2012). Stimulus transients or saccadic eye movements may be able to reset the phase of these rhythms, or saccades may be triggered to occur at fixed phases of attentional oscillations (Hogendoorn, 2016; Landau & Fries, 2012). Given the many similarities between saccades and eye blinks and the transients triggered by blinks, it is possible that eye blinks cause a phase reset for such rhythms or occur at a specific phase. In this view, the early and late windows with performance enhancements after eye blinks identified above might be the consequence of synchronized performance fluctuations. Unfortunately, the RSVP paradigm employed in our study does not lend itself to an analysis of behavioral performance rhythms time-locked to the blink. There are insufficient numbers of trials per participant to analyze performance robustly in the frequency domain.

The postblink boost may be consistent with the view that both visual attention and eye blinks are related to cortical dopaminergic mechanisms. Several studies found that increasing dopamine levels lead to increased spontaneous blink rates (Karson, 1983; Kleven & Koek, 1996). Furthermore, infusing dopamine D1 receptors in monkeys’ frontal eye fields (FEFs) with D1-receptor agonist resulted in monkeys being more likely to choose stimuli targets that fell within the corresponding FEF response field (Noudoost & Moore, 2011), highlighting the role of dopamine in influencing attention. It is plausible that dopamine levels influence both eye blink generation and attentional modulation.

Some low-level, optical effects of eye blinks should be considered, as they might also bear on perceptual abilities as measured in the current experiments. Blinks are known to induce changes in pupil size as the eye adapts to different light levels during and after the lid closure (Winn, Whitaker, Elliott, & Phillips, 1994). Pupil sizes fluctuate in a prototypical pattern after blinks and may thus affect perceptual properties of a viewed stimulus. Examining pupil size in our data revealed no obvious relationship between pupil sizes and performance (see Supplementary Figure S1). VB and AB have distinct but consistent pupil size change patterns across all experiments. Yet, changes in behavioral performance did not follow similar time courses.

Another low-level effect of eye blinks—and some might argue their primary physiological function—is the renewal of the tear film layer over the cornea, which aids refraction and increases the acuity of vision (Montés-Micó, 2007; Montés-Micó, Cervino, Ferrer-Blasco, García-Lázaro, & Madrid-Costa, 2010). However, the refractive properties of the corneal surface as a function of time after a blink are optimal at about 6 s after a blink (Montés-Micó, Alió, & Charman, 2005), which is outside of the time scale we examined here.

Blinks can be categorized as voluntary (consciously initiated), spontaneous (unconsciously initiated), or reflexive (initiated by foreign objects). Eye blinks in the current study were all voluntary, potentially limiting the generalizability of our results to other situations. However, some studies have argued that all types of blinks share similar mechanisms to a certain extent. For example, suppression of detection performance after blinks is similar for reflexive and voluntary blinks (Manning et al., 1983). Blink adaptation, in which position changes of a fixation target across a blink lead to adaptive gaze shifts, occurs similarly for spontaneous, voluntary, and reflexive blinks (Lau & Maus, 2019). Postblink neural transient characteristics in Golan et al. (2016), too, are somewhat similar between voluntary and spontaneous blinks. Thus, the behavioral consequences of voluntary eye blinks could be similar to spontaneous eye blinks to some extent. At the very least, it is interesting to note that in the case of voluntary blinks, perceptual performance can be modulated by eye blinks, and the resulting boost appears over and above effects of visual transients.

The timing of eye blinks can occur strategically during times of lower target probability (Hoppe et al., 2018), and in a more ecological situation of watching a video, blinks occur at highly consistent time points not overlapping with meaningful actions (Nakano et al., 2009). Both these findings highlight the importance of suppressing blinks during behaviorally relevant time periods, since obviously our eyes should remain open when relevant information is present. However, our results suggest that in situations where the timing of relevant events is predictable during intentional visual search, it might be beneficial to blink just before an event.

## Conclusions

Our results showed that eye blinks are accompanied by subsequent boosts in performance in object recognition tasks. This sheds light on how eye blinks can influence visual performance apart from the obvious occlusion of the visual field and the consequences of blinks for spatial and temporal visual attention.

## Supplementary Material

Supplement 1
