# Supplementary material for: Boosted visual performance after eye blinks
Source: J Vis. 2020 Oct 1;20(10):2. doi: 10.1167/jov.20.10.2 (PMC7545084; doi:10.1167/jov.20.10.2)
Supplement: Supplement 1 [file jovi-20-10-2_s001.docx]

**Supplementary Information**


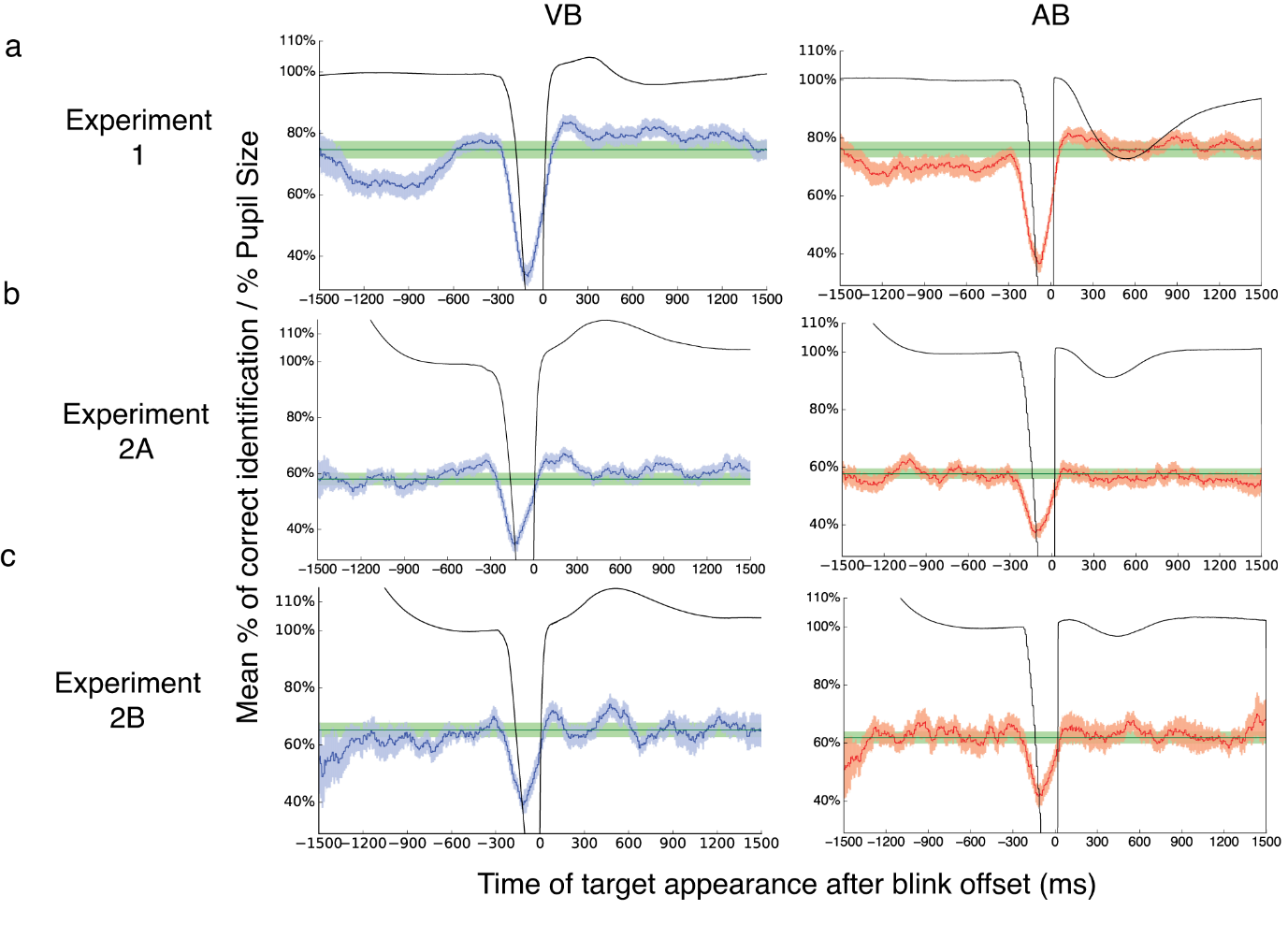


***Figure S1.*** ***Performance plotted against pupil size.*** Performance, as in Figure 2, plotted with a rolling average window of 200 ms on each millisecond on the x-axis, with bootstrapped standard error of mean (shaded areas). Additionally, averaged time courses of pupil size (normalized to the pre-blink pupil size at -250 ms to -50 ms before blink onset) are shown by the black lines. Pupil normalized size above 110% are not shown. The pupil size drops at the beginning of the time courses in Experiment 2 were due to the sudden appearance of a bigger stimuli at trial onset. Each condition was plotted against their own respective control (that was held on the same day). Control performance across sessions generally tended to be consistent. **a.** Experiment 1. **b.** Experiment 2A. **c.** Experiment 2B.

**
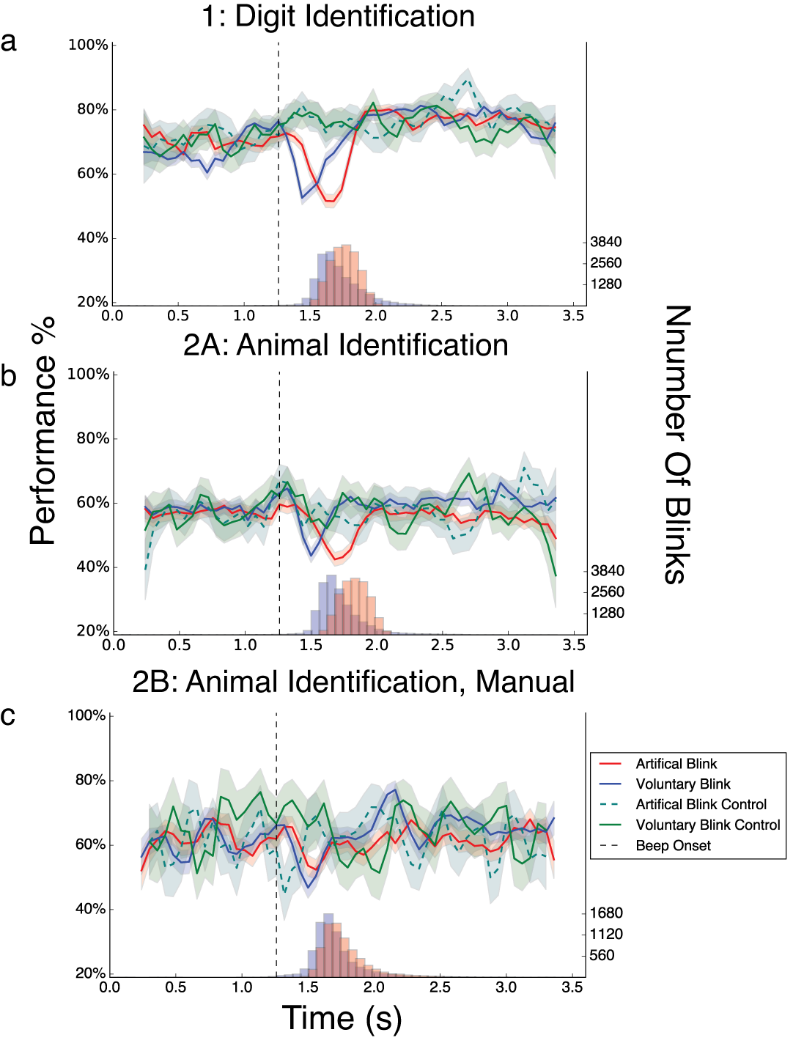
**

***Figure S2.*** Performance for all experiments as in Figure 2, but time locked to the beginning of the trial. The histograms at the bottom of each panel show the number of trials with a blink ending in this bin. Bins with less than 15 trials (usually the first and last few bins) are not shown. The vertical dashed line shows the time of the auditory beep that cued participants to blink (at 1.26 s). Here, the control conditions run in each session are plotted separately (the grand average of no-blink controls is plotted in Figure 2). **a.** Experiment 1. **b.** Experiment 2A **c.** Experiment 2B.
